# *In Vitro* Synergistic Activity of Antimicrobial Agents in Combination against Clinical Isolates of Colistin-Resistant Acinetobacter baumannii

**DOI:** 10.1128/AAC.00839-16

**Published:** 2016-10-21

**Authors:** Seongman Bae, Min-Chul Kim, Su-Jin Park, Hee Sueng Kim, Heungsup Sung, Mi-Na Kim, Sung-Han Kim, Sang-Oh Lee, Sang-Ho Choi, Jun Hee Woo, Yang Soo Kim, Yong Pil Chong

**Affiliations:** a Department of Infectious Diseases, Asan Medical Center, University of Ulsan College of Medicine, Seoul, Republic of Korea; b Department of Laboratory Medicine, Asan Medical Center, University of Ulsan College of Medicine, Seoul, Republic of Korea

## Abstract

Emerging resistance to colistin in clinical Acinetobacter baumannii isolates is of growing concern. Since current treatment options for these strains are extremely limited, we investigated the *in vitro* activities of various antimicrobial combinations against colistin-resistant A. baumannii. Nine clinical isolates (8 from bacteremia cases and 1 from a pneumonia case) of colistin-resistant A. baumannii were collected in Asan Medical Center, Seoul, South Korea, between January 2010 and December 2012. To screen for potential synergistic effects, multiple combinations of two antimicrobials among 12 commercially available agents were tested using the multiple-combination bactericidal test (MCBT). Checkerboard tests were performed to validate these results. Among the 9 colistin-resistant strains, 6 were pandrug resistant and 3 were extensively drug resistant. With MCBT, the most effective combinations were colistin-rifampin and colistin-teicoplanin; both combinations showed synergistic effect against 8 of 9 strains. Colistin-aztreonam, colistin-meropenem, and colistin-vancomycin combinations showed synergy against seven strains. Colistin was the most common constituent of antimicrobial combinations that were active against colistin-resistant A. baumannii. Checkerboard tests were then conducted in colistin-based combinations. Notably, colistin-rifampin showed synergism against all nine strains (100%). Both colistin-vancomycin and colistin-teicoplanin showed either synergy or partial synergy. Colistin combined with another β-lactam agent (aztreonam, ceftazidime, or meropenem) showed a relatively moderate effect. Colistin combined with ampicillin-sulbactam, tigecycline, amikacin, azithromycin, or trimethoprim-sulfamethoxazole demonstrated limited synergism. Using MCBT and checkerboard tests, we found that only colistin-based combinations, particularly those with rifampin, glycopeptides, or β-lactams, may confer therapeutic benefits against colistin-resistant A. baumannii.

## INTRODUCTION

Acinetobacter baumannii is regarded as an important nosocomial pathogen causing various infections, including ventilator-associated pneumonia, bloodstream infections, surgical site infections, and urinary tract infections (1). It has become more problematic by developing resistance to a wide range of antimicrobials, including carbapenems (2–5). Colistin, the most active agent against multidrug-resistant (MDR) Gram-negative pathogens *in vitro*, has been reintroduced for the treatment of carbapenem-resistant A. baumannii (6). Unfortunately, colistin-resistant A. baumannii strains have been reported recently (7). As these strains are simultaneously resistant to most antimicrobial agents, treatment options for them are extremely limited (8). A few previous studies evaluated the *in vitro* synergism of antimicrobial combinations against colistin-resistant A. baumannii (9–11). In those studies, however, the number of antimicrobial agents tested did not exceed four, and only colistin-based combinations were tested. In real clinical practice, colistin-associated nephrotoxicity occurs in about 40% of treated patients, and colistin therapy is frequently stopped because of this (8, 12, 13). Therefore, the *in vitro* efficacy of non-colistin-based combinations against colistin-resistant A. baumannii strains should also be evaluated. The aim of this study was to assess the *in vitro* efficacy of antimicrobial combinations, among 12 commercially available antimicrobial agents, against clinical isolates of colistin-resistant A. baumannii using the multiple-combination bactericidal test (MCBT) and checkerboard method.

## MATERIALS AND METHODS

### Patients, bacterial isolates, and selection of antimicrobial agents.

Patients infected with colistin-resistant A. baumannii were identified at the Asan Medical Center, Seoul, South Korea, between January 2010 and December 2012. Colistin susceptibility testing was performed on all blood and some sputum isolates at the request of the treating physician. A colistin MIC of >2 mg/liter indicated resistance (14). Nine representative colistin-resistant A. baumannii isolates from different patients were included in this study. The clinical data of these patients were collected from electronic medical records, and A. baumannii was identified using a MicroScan system (Dade Behring, Deerfield, IL, USA) and/or a Vitek 2 system (bioMérieux Inc., La Balme les Grottes, France). The following 12 antimicrobial agents were selected based on previous studies suggesting their antimicrobial efficacy against MDR A. baumannii: colistin, ampicillin-sulbactam, amikacin, azithromycin, aztreonam, ceftazidime, meropenem, rifampin, tigecycline, trimethoprim-sulfamethoxazole, vancomycin, and teicoplanin (15–27).

### Susceptibility testing and interpretation.

*In vitro* antimicrobial susceptibility testing was performed in triplicate using the broth microdilution method according to the Clinical and Laboratory Standards Institute (CLSI) guidelines (14). Fresh Mueller-Hinton broth was used for all susceptibility testing. CLSI susceptibility criteria were used, except with azithromycin, aztreonam, vancomycin, teicoplanin, tigecycline, and rifampin. No susceptibility breakpoints for rifampin and tigecycline are given in the CLSI guidelines; therefore, CLSI criteria recommended for staphylococci were applied to rifampin (MIC ≥ 4 mg/liter as resistance), and European Committee on Antimicrobial Susceptibility Testing (EUCAST) criteria for Enterobacteriaceae were used for tigecycline (MIC > 2 mg/liter as resistance) (28). Escherichia coli ATCC 25922 was used as a reference strain, and all results determined with this strain were within the CLSI quality control ranges. The category of extensively drug-resistant (XDR) strains was defined as nonsusceptibility to at least one agent in all but two or fewer antimicrobial categories, and pandrug-resistant (PDR) was defined as nonsusceptibility to all antimicrobial agents (29).

### Detection of OXA genes and genes encoding metallo-β-lactamases.

The presence of a variety of carbapenemase genes (OXA-23, -48, -50, -51, -58, -60, -69, IMP-1, IMP-2, VIM-1, VIM-2, GIM-1, SPM-1, and SIM-1 genes) was evaluated by PCR with specific primers (30). PCR products were then sequenced and analyzed using the NCBI BLAST program.

### Molecular typing by MLST.

Multilocus sequence typing (MLST) was performed on seven housekeeping genes (*gltA*, *gyrB*, *gdhB*, *recA*, *cpn60*, *gpi*, and *rpoD*) as described previously (31). Isolates were assigned to sequence types (STs) using tools available on the A. baumannii MLST database (http://pubmlst.org/abaumannii/).

### MCBT.

The multiple-combination bactericidal test (MCBT) was performed to test combinations of two antimicrobials as previously described (32–35). Combinations of two antimicrobials were placed in 96-well, round-bottomed microtiter plates (Nunc Inc., Roskilde, Denmark). The antimicrobial agents were prepared in Mueller-Hinton II cation-adjusted broth (MHB II; Becton, Dickinson Microbiology Systems, Cockeysville, MD) at 10 times the required concentrations. One or two antimicrobial agents were added, each in 10-μl volumes, to the wells. The necessary volume of MHB II was then added to the wells containing antimicrobial agents. The A. baumannii inocula consisted of 70 μl of a 100-fold dilution of a 0.5 McFarland turbidity standard prepared during the growth phase of culture in tryptone soya broth (Oxoid Laboratories, Basingstoke, United Kingdom). The final inoculum concentration was 5 × 10^5^ CFU/ml in each well. Growth and sterility control wells (no antibiotic and no inoculum, respectively) were included in all plates. Plates were incubated at 35°C for 48 h. At 24 and 48 h, the wells were examined for turbidity. Each well with no visible growth at 48 h was subcultured to establish whether 99.9% killing was achieved. Reproducibility of the MCBT results was confirmed in triplicate. For the purposes of the MCBT analysis, combinations were considered synergistic if bactericidal activity (99.9% killing) was achieved when the two agents were tested in combination.

The final concentrations of antimicrobials selected for MCBT corresponded to the criteria for resistance (35). The antimicrobial agents were used in MCBT at the following fixed concentrations: colistin at 2 mg/liter, ampicillin-sulbactam at 16/8 mg/liter, amikacin at 16 mg/liter, azithromycin at 4 mg/liter, aztreonam at 16 mg/liter, ceftazidime at 16 mg/liter, meropenem at 8 mg/liter, rifampin at 2 mg/liter, tigecycline at 2 mg/liter, trimethoprim-sulfamethoxazole at 4/76 mg/liter, vancomycin at 4 mg/liter, and teicoplanin at 16 mg/liter.

### Synergy testing of colistin combinations with the checkerboard method.

To identify synergistic effects, the checkerboard synergy test was performed in triplicate in 96-well microtiter plates containing colistin and 1 of 11 other antimicrobials. Each antimicrobial was diluted using an automated dilutor, with concentrations ranging from 0.031× MIC to 4× MIC. The initial inoculum was approximately 5 × 10^5^ CFU/ml. Microtiter trays were incubated at 35°C for 48 h under aerobic conditions (36).

After incubation, any well showing turbidity was considered to exhibit microbiological growth. The fractional inhibitory concentration index (FICI) was calculated for each antibiotic in each combination. The mean FICI of all nonturbid wells, along the turbidity/nonturbidity interface, was then calculated (37). The FICI results for each combination against each test isolate were interpreted as follows: FICI of ≤0.5, synergism; FICI of between 0.5 and 1, partial synergism; FICI of ≥1 but <4, indifference; FICI of ≥4, antagonism (38, 39).

## RESULTS

### Microbiological and genotypic characteristics of colistin-resistant A. baumannii.

Of nine colistin-resistant A. baumannii strains, eight were blood isolates and one was a sputum isolate. All of the strains were also resistant to carbapenems. Results of MLST, carbapenemase types, and MICs of antimicrobials against each strain are summarized in Table 1 and in Table S1 in the supplemental material. All of the tested strains carried the OXA-51 gene, and OXA-23 was detected in seven strains (78%). Eight of nine strains had the IMP-1 gene encoding a metallo-β-lactamase. By MLST, 7 strains were found to belong to ST191, while the remaining two were ST357. Six of nine strains were resistant to all classes of antimicrobials (PDR), and the remaining three A. baumannii strains were XDR.

**TABLE 1:** The MIC values of antimicrobial agents against colistin-resistant Acinetobacter baumannii strains^*a*^

Strain	MIC (μg/ml)^*a*^											
	CST	SAM	TGC	AMK	AZM	ATM	CAZ	MEM	RIF	SXT	VAN	TEC
a	256	64/32	8	1,024	>128	64	128	64	4	64/1,216	256	512
b	256	64/32	4	1,024	>128	128	128	64	8	32/608	512	256
c	16	64/32	4	4	4	128	64	64	8	32/608	256	256
d	1,024	32/16	4	>4,096	>128	64	128	64	4	32/608	512	256
e	8	32/16	32	8	32	64	512	64	8	2/38	512	512
f	64	1,024/512	16	1,024	>128	1,024	64	256	16	32/608	512	256
g	16	32/16	32	1,024	>128	64	64	64	8	128/2,432	512	128
h	8	16/8	4	4	>128	64	128	32	8	2/38	256	128
i	1,024	128/64	4	512	>128	128	128	64	256	32/608	256	128

a Abbreviations: CST, colistin; SAM, ampicillin-sulbactam; TGC, tigecycline; AMK, amikacin; AZM, azithromycin; ATM, aztreonam; CAZ, ceftazidime; MEM, meropenem; RIF, rifampin; SXT, trimethoprim-sulfamethoxazole; VAN, vancomycin; TEC, teicoplanin.

### MCBT.

Using the MCBT method, each two-drug combination was tested (Table 2). The most effective combination regimens were colistin-rifampin and colistin-teicoplanin, both of which showed synergy against eight of nine strains. The colistin-aztreonam, colistin-meropenem, and colistin-vancomycin combinations were synergistic against seven strains. All of the regimens exhibiting synergistic effect against at least four strains included colistin. Other combinations were active against two or fewer strains. Among the colistin-based combinations, only colistin-tigecycline was not synergistic against any of the strains tested.

**TABLE 2:** Combined effects of 12 antimicrobial drugs on nine colistin-resistant A. baumannii strains in the multiple-combination bactericidal test

Agents^*a*^	Strain(s) killed^*b*^
SAM + RIF	e
SAM + SXT	f
SAM + TEC	d
AMK + CAZ	f
AMK + SXT	f
AZM + CAZ	f
AZM + SXT	f
AZM + TEC	e
ATM + CAZ	g
ATM + SXT	f
ATM + TEC	e
CAZ + MEM	f
CAZ + RIF	f
CAZ + TGC	f
CAZ + SXT	f
CAZ + VAN	f
MEM + RIF	h
MEM + SXT	f
MEM + TEC	e
RIF + SXT	f
SXT + VAN	f
AMK + RIF	a,f
CAZ + TEC	e,f
CST + AZM	b, d, e, h
CST + AMK	b, d, f, g
CST + SXT	b, d, f, h
CST + SAM	b, c, d, e, g
CST + CAZ	b, c, e, f, g, h
CST + ATM	a, b, c, d, e, g, h, i
CST + MEM	a, b, c, d, e, g, h, i
CST + VAN	a, b, c, d, e, g, h, i
CST + TEC	a, b, c, d, e, f, g, h, i (all)
CST + RIF	a, b, c, d, e, f, g, h, i (all)

a Other antimicrobial combinations that are not shown (e.g., CST + TGC) were not synergistic against any of the strains tested.

b If an XDR strain (c, e, or h) was killed because the drug MIC for the strain was equal to or lower than the tested concentration of an antimicrobial agent, in an antimicrobial combination that included this agent, the strain was not listed.

### Checkerboard synergy test.

Since only colistin-based regimens were highly effective in the MCBT, checkerboard tests were performed to validate presence of synergism among these combination regimens. As shown in Table 3, results of the checkerboard synergy analysis of colistin-resistant A. baumannii were similar to those of MCBT. The colistin-rifampin combination was fully synergistic against nine of the A. baumannii strains tested. The combinations of colistin-vancomycin and colistin-teicoplanin showed either synergy or partial synergy against all strains. However, colistin-vancomycin (6/9, 67%) was more frequently synergistic than colistin-teicoplanin (4/9, 45%). With colistin-aztreonam and colistin-ceftazidime, and with colistin-meropenem, 7 (78%) strains exhibited synergy and partial synergy, respectively. Colistin combinations with ampicillin-sulbactam, tigecycline, azithromycin, and trimethoprim-sulfamethoxazole were synergistic against only one strain. Colistin-tigecycline and colistin-azithromycin showed indifference against seven and eight strains, respectively. No antagonistic interactions were observed with any of the combinations evaluated.

**TABLE 3:** Results of the checkerboard synergy test of nine strains of colistin-resistant A. baumannii^*a*^

Agents	Strain(s) with the indicated test result		
	Synergistic (FICI ≤ 0.5)	Partially synergistic (0.5 < FICI < 1)	Indifferent (1 ≤ FICI < 4)
CST + TGC	h	f	a, b, c, d, e, g, i
CST + AZM	f	-	a, b, c, d, e, g, h, i
CST + AMK	f, g, h	-	a, b, c, d, e, i
CST + SXT	f	a, g, h	b, c, d, e, i
CST + SAM	h	b, d, f, g, i	a, c, e
CST + CAZ	a, f, g, h	b, c, d	e, i
CST + ATM	a, b, d, i	c, g, h	e, f
CST + MEM	e, g, h	a, b, d, f	c, i
CST + TEC	a, e, f, i	b, c, d, g, h	-
CST + VAN	a, b, d, e, f, g, h	c, i	-
CST + RIF	a, b, c, d, e, f, g, h, i	-	-

a Abbreviation: FICI, fractional inhibitory concentration index.

### Clinical characteristics and treatment outcomes.

The clinical characteristics and treatment outcomes of patients with colistin-resistant A. baumannii infections are summarized in Table 4. Most patients had severe underlying diseases, such as malignancy, hematologic disease, liver transplantation, and acute liver failure related to a hepatitis B virus (HBV) flare-up. All nine patients were nosocomially infected with A. baumannii, and 7 of 9 patients experienced an intensive care unit (ICU) stay. Four of the nine patients had a history of prior colistin use, and all of the patients had previously used carbapenems. Antibiotic regimens and empirical treatment outcomes varied by patient. Three patients were treated with colistin-based combinations, and microbiological eradication was achieved in two patients. The mortality rate was high, and most patients (67%) died within 14 days.

**TABLE 4:** Clinical characteristics and treatment outcomes of patients with colistin-resistant A. baumannii infection^*a*^

Variable	Result(s) for patient:								
	a	b	c	d	e	f	g	h	i
Age (yr)/gender	61/M	33/M	66/M	51/F	82/M	43/F	51/M	69/F	67/F
Underlying disease	CBD cancer	LT	Hepatocellular carcinoma	Fulminant hepatitis due to HBV flare-up	Colon cancer, pelvic abscess	Myelodysplastic syndrome on BMT	LT	Metastatic CBD cancer	Supraglottic cancer
Acquisition	Hospital onset	Hospital onset	Hospital onset	Hospital onset	Hospital onset	Hospital onset	Hospital onset	Hospital onset	Hospital onset
Ward	SICU	SICU	SICU	MICU	SICU	BMT unit	LT unit	General ward	MICU
Type of infection	VAP, bacteremia	cIAI, bacteremia	cIAI, bacteremia	HAP, bacteremia	HAP, bacteremia	primary bacteremia	primary bacteremia	HAP, bacteremia	VAP
Clinical status									
Previous use of colistin	Yes	Yes	No	No	No	No	No	Yes	Yes
Previous use of carbapenem	Yes	Yes	Yes	Yes	Yes	Yes	Yes	Yes	Yes
Recent operation	Yes	Yes	Yes	No	Yes	No	Yes	No	Yes
Antibiotic therapy	Colistin, vancomycin, amikacin	Colistin, meropenem, vancomycin	Colistin, vancomycin	Meropenem, vancomycin, levofloxacin	Meropenem, vancomycin, metronidazole	Imipenem, vancomycin, levofloxacin	Linezolid, levofloxacin	Tigecycline	Tigecycline, teicoplanin, rifampin, ampicillin-sulbactam
Microbiological eradication	Yes	No	Yes	No	No	No	Yes	No	No
Mortality									
14 day	Yes	No	Yes	Yes	Yes	Yes	No	Yes	No
28 day	Yes	No	Yes	Yes	Yes	Yes	No	Yes	No
In hospital	Yes	Yes	Yes	Yes	Yes	Yes	No	Yes	No
Infection related	No	No	No	Yes	Yes	Yes	No	Yes	No

a Abbreviations: M, male; F, female; CBD, common bile duct; LT, liver transplantation; BMT, bone marrow transplantation; SICU, surgical intensive care unit; MICU, medical intensive care unit; VAP, ventilator-associated pneumonia; cIAI, complicated intra-abdominal infection; HAP, hospital-acquired pneumonia.

## DISCUSSION

The main purpose of this study was to assess the *in vitro* synergistic effects of antimicrobial combinations against colistin-resistant A. baumannii. Combinations of commonly used antimicrobial agents were tested by MCBT, and synergistic results were confirmed using the checkerboard method. By MCBT, colistin was determined to be the most common constituent of antimicrobial combinations that were active against colistin-resistant A. baumannii. Non-colistin-based combinations were not active against these strains. Colistin-rifampin or colistin-cell wall active agent combinations showed synergistic effects against most strains by the checkerboard test. The results of colistin-based combinations with meropenem, rifampin, aztreonam, ceftazidime, teicoplanin, and vancomycin in MCBT were generally concordant with those of the checkerboard test. Hence, in daily clinical practice, a stepwise approach using MCBT can be applied to choose the best antimicrobial combination for colistin-resistant A. baumannii if other reliable but labor-intensive synergy tests such as the checkerboard and time-kill methods are not available. We may choose a specific antimicrobial combination according to results of growth inhibition at 48 h on MCBT; we can then further confirm or modify the regimen by checking 99.9% killing.

Hypothetically, colistin-resistant A. baumannii may have a modified outer membrane, which can increase permeability with respect to cell wall-targeted antimicrobial agents. Two previous studies reported that colistin-resistant A. baumannii strains had higher susceptibility rates for the majority of antimicrobial agents than colistin-susceptible strains (40, 41). In contrast, antimicrobial agents showed high MICs against colistin-resistant strains in the current study and the recent study by Qureshi et al. (8). These differences were probably due to frequent simultaneous exposure to carbapenems, vancomycin, and colistin.

Colistin with rifampin has been the most frequently studied combination *in vitro* (7). Although a recent randomized clinical trial failed to show a difference in outcomes between colistin-rifampin and colistin monotherapies against XDR A. baumannii, the microbiological eradication rate was significantly higher in the combination arm (42). In the present study, a strong synergistic effect from colistin combined with rifampin was shown in both the MCBT and the checkerboard test. Notably, with the checkerboard test, colistin-rifampin was found to be fully synergistic (FICI ≤ 0.5) against all nine (100%) A. baumannii strains. Therefore, the clinical efficacy of colistin-rifampin should be further evaluated in colistin-resistant A. baumannii infections.

Glycopeptide MICs of tested strains were higher than those of two previous studies indicating relatively low MICs of glycopeptides against colistin-resistant A. baumannii (43, 44). Albeit with high MICs against our strains, vancomycin and teicoplanin consistently showed synergism in combination with colistin, in accordance with previous *in vitro* and *in vivo* studies (27, 43, 44). We conjectured that glycopeptides might be effective in combination with colistin, regardless of its MIC, because of an adjuvant permeabilizing effect of colistin on the A. baumannii outer membrane. In this regard, other cell wall-active agents such as ceftazidime, aztreonam, and meropenem also tended to show synergistic effects in our tests.

Tigecycline, regarded as an effective treatment option for MDR A. baumannii infections, showed low antimicrobial activity against colistin-resistant strains in the present study. Tigecycline-containing combinations did not show synergistic effect against any of the strains in MCBT, even in combination with colistin. Colistin-tigecycline showed only limited synergistic effects by the checkerboard test. Cheng et al. reported a higher adjusted 14-day mortality rate in the colistin-tigecycline combination treatment group than in the colistin-carbapenem treatment group in one prospective, observational study of XDR A. baumannii bacteremia (45). They deduced that tigecycline was less effective because this agent targets the 30S ribosomal subunit, not the cell wall.

Our study had several limitations. All tested strains were collected from a single tertiary center, and the mechanism of colistin resistance was not evaluated, which limits our ability to generalize from these results. However, results of the synergy tests performed on study strains were similar to those of previous colistin-based studies. In addition, FICIs from the checkerboard test can differ, depending on the various methods used for interpretation (46). Finally, this was an *in vitro* study that did not test clinical outcomes; clinical studies are needed to confirm our findings.

In conclusion, using MCBT and checkerboard testing, we found that only colistin-based combinations, particularly combinations with rifampin, glycopeptides, or β-lactams, should be expected to confer therapeutic benefits in colistin-resistant A. baumannii infections. The development of new antimicrobial agents is urgently needed to treat infections by this pathogen.

## Supplementary Material

Supplemental material

## References

[1] Peleg AY, Seifert H, Paterson DL 2008 Acinetobacter baumannii: emergence of a successful pathogen. Clin Microbiol Rev 21:538–582. doi:10.1128/CMR.00058-07. 18625687 PMC2493088

[2] Vila J, Pachon J 2012 Therapeutic options for Acinetobacter baumannii infections: an update. Expert Opin Pharmacother 13:2319–2336. doi:10.1517/14656566.2012.729820. 23035697

[3] Fishbain J, Peleg AY 2010 Treatment of Acinetobacter infections. Clin Infect Dis 51:79–84. doi:10.1086/653120. 20504234

[4] Munoz-Price LS, Weinstein RA 2008 Acinetobacter infection. N Engl J Med 358:1271–1281. doi:10.1056/NEJMra070741. 18354105

[5] Fournier PE, Vallenet D, Barbe V, Audic S, Ogata H, Poirel L, Richet H, Robert C, Mangenot S, Abergel C, Nordmann P, Weissenbach J, Raoult D, Claverie JM 2006 Comparative genomics of multidrug resistance in Acinetobacter baumannii. PLoS Genet 2:e7. doi:10.1371/journal.pgen.0020007. 16415984 PMC1326220

[6] Li J, Nation RL, Turnidge JD, Milne RW, Coulthard K, Rayner CR, Paterson DL 2006 Colistin: the re-emerging antibiotic for multidrug-resistant Gram-negative bacterial infections. Lancet Infect Dis 6:589–601. doi:10.1016/S1473-3099(06)70580-1. 16931410

[7] Cai Y, Chai D, Wang R, Liang B, Bai N 2012 Colistin resistance of Acinetobacter baumannii: clinical reports, mechanisms and antimicrobial strategies. J Antimicrob Chemother 67:1607–1615. doi:10.1093/jac/dks084. 22441575

[8] Qureshi ZA, Hittle LE, O'Hara JA, Rivera JI, Syed A, Shields RK, Pasculle AW, Ernst RK, Doi Y 2015 Colistin-resistant Acinetobacter baumannii: beyond carbapenem resistance. Clin Infect Dis 60:1295–1303. 25632010 10.1093/cid/civ048 PMC4462660

[9] Nastro M, Rodriguez CH, Monge R, Zintgraff J, Neira L, Rebollo M, Vay C, Famiglietti A 2014 Activity of the colistin-rifampicin combination against colistin-resistant, carbapenemase-producing Gram-negative bacteria. J Chemother 26:211–216. doi:10.1179/1973947813Y.0000000136. 24070502

[10] Percin D, Akyol S, Kalin G 2014 *In vitro* synergism of combinations of colistin with selected antibiotics against colistin-resistant Acinetobacter baumannii. GMS Hyg Infect Control 9:Doc14. 25152859 10.3205/dgkh000234 PMC4141631

[11] Vidaillac C, Benichou L, Duval RE 2012 *In vitro* synergy of colistin combinations against colistin-resistant Acinetobacter baumannii, Pseudomonas aeruginosa, and Klebsiella pneumoniae isolates. Antimicrob Agents Chemother 56:4856–4861. doi:10.1128/AAC.05996-11. 22751540 PMC3421898

[12] Pogue JM, Lee J, Marchaim D, Yee V, Zhao JJ, Chopra T, Lephart P, Kaye KS 2011 Incidence of and risk factors for colistin-associated nephrotoxicity in a large academic health system. Clin Infect Dis 53:879–884. doi:10.1093/cid/cir611. 21900484

[13] Hartzell JD, Neff R, Ake J, Howard R, Olson S, Paolino K, Vishnepolsky M, Weintrob A, Wortmann G 2009 Nephrotoxicity associated with intravenous colistin (colistimethate sodium) treatment at a tertiary care medical center. Clin Infect Dis 48:1724–1728. doi:10.1086/599225. 19438394

[14] Clinical and Laboratory Standards Institute. 2014 Performance standards for antimicrobial susceptibility testing: 24th informational supplement M100-S24. Clinical and Laboratory Standards Institute, Wayne, PA.

[15] Sopirala MM, Mangino JE, Gebreyes WA, Biller B, Bannerman T, Balada-Llasat JM, Pancholi P 2010 Synergy testing by Etest, microdilution checkerboard, and time-kill methods for pan-drug-resistant Acinetobacter baumannii. Antimicrob Agents Chemother 54:4678–4683. doi:10.1128/AAC.00497-10. 20713678 PMC2976112

[16] Kiffer CR, Sampaio JL, Sinto S, Oplustil CP, Koga PC, Arruda AC, Turner PJ, Mendes C 2005 *In vitro* synergy test of meropenem and sulbactam against clinical isolates of Acinetobacter baumannii. Diagn Microbiol Infect Dis 52:317–322. doi:10.1016/j.diagmicrobio.2005.03.003. 15936166

[17] Moland ES, Craft DW, Hong SG, Kim SY, Hachmeister L, Sayed SD, Thomson KS 2008 *In vitro* activity of tigecycline against multidrug-resistant Acinetobacter baumannii and selection of tigecycline-amikacin synergy. Antimicrob Agents Chemother 52:2940–2942. doi:10.1128/AAC.01581-07. 18519722 PMC2493109

[18] Timurkaynak F, Can F, Azap OK, Demirbilek M, Arslan H, Karaman SO 2006 *In vitro* activities of non-traditional antimicrobials alone or in combination against multidrug-resistant strains of Pseudomonas aeruginosa and Acinetobacter baumannii isolated from intensive care units. Int J Antimicrob Agents 27:224–228. doi:10.1016/j.ijantimicag.2005.10.012. 16464562

[19] Fernández Cuenca F, Pascual A, Martínez Martínez L, Perea EJ 2003 *In vitro* activity of azithromycin against clinical isolates of Acinetobacter baumannii. Rev Esp Quimioter 16:204–208. (In Spanish.) 12973458

[20] Malone L, Kwon DH 2013 Carbapenem-associated multidrug-resistant Acinetobacter baumannii are sensitised by aztreonam in combination with polyamines. Int J Antimicrob Agents 41:70–74. doi:10.1016/j.ijantimicag.2012.08.009. 23148986 PMC3530022

[21] Drago L, De Vecchi E, Nicola L, Colombo A, Guerra A, Gismondo MR 2004 Activity of levofloxacin and ciprofloxacin in combination with cefepime, ceftazidime, imipenem, piperacillin-tazobactam and amikacin against different Pseudomonas aeruginosa phenotypes and Acinetobacter spp. Chemotherapy 50:202–210. doi:10.1159/000081033. 15452399

[22] Pankey GA, Ashcraft DS 2009 The detection of synergy between meropenem and polymyxin B against meropenem-resistant Acinetobacter baumannii using Etest and time-kill assay. Diagn Microbiol Infect Dis 63:228–232. doi:10.1016/j.diagmicrobio.2008.11.002. 19150711

[23] Tripodi MF, Durante-Mangoni E, Fortunato R, Utili R, Zarrilli R 2007 Comparative activities of colistin, rifampicin, imipenem and sulbactam/ampicillin alone or in combination against epidemic multidrug-resistant Acinetobacter baumannii isolates producing OXA-58 carbapenemases. Int J Antimicrob Agents 30:537–540. doi:10.1016/j.ijantimicag.2007.07.007. 17851050

[24] Sheng WH, Wang JT, Li SY, Lin YC, Cheng A, Chen YC, Chang SC 2011 Comparative *in vitro* antimicrobial susceptibilities and synergistic activities of antimicrobial combinations against carbapenem-resistant Acinetobacter species: Acinetobacter baumannii versus Acinetobacter genospecies 3 and 13TU. Diagn Microbiol Infect Dis 70:380–386. doi:10.1016/j.diagmicrobio.2011.03.003. 21558048

[25] Chuang YC, Cheng CY, Sheng WH, Sun HY, Wang JT, Chen YC, Chang SC 2014 Effectiveness of tigecycline-based versus colistin-based therapy for treatment of pneumonia caused by multidrug-resistant Acinetobacter baumannii in a critical setting: a matched cohort analysis. BMC Infect Dis 14:102. doi:10.1186/1471-2334-14-102. 24564226 PMC3936940

[26] Falagas ME, Vardakas KZ, Roussos NS 2015 Trimethoprim/sulfamethoxazole for Acinetobacter spp.: a review of current microbiological and clinical evidence. Int J Antimicrob Agents 46:231–241. doi:10.1016/j.ijantimicag.2015.04.002. 26070662

[27] Gordon NC, Png K, Wareham DW 2010 Potent synergy and sustained bactericidal activity of a vancomycin-colistin combination versus multidrug-resistant strains of Acinetobacter baumannii. Antimicrob Agents Chemother 54:5316–5322. doi:10.1128/AAC.00922-10. 20876375 PMC2981237

[28] European Committee on Antimicrobial Susceptibility Testing (EUCAST) Steering Committee. 2006 EUCAST technical note on tigecycline. Clin Microbiol Infect 12:1147–1149. doi:10.1111/j.1469-0691.2006.01578.x. 17063597

[29] Magiorakos AP, Srinivasan A, Carey RB, Carmeli Y, Falagas ME, Giske CG, Harbarth S, Hindler JF, Kahlmeter G, Olsson-Liljequist B, Paterson DL, Rice LB, Stelling J, Struelens MJ, Vatopoulos A, Weber JT, Monnet DL 2012 Multidrug-resistant, extensively drug-resistant and pandrug-resistant bacteria: an international expert proposal for interim standard definitions for acquired resistance. Clin Microbiol Infect 18:268–281. doi:10.1111/j.1469-0691.2011.03570.x. 21793988

[30] Queenan AM, Bush K 2007 Carbapenemases: the versatile beta-lactamases. Clin Microbiol Rev 20:440–458, table of contents. doi:10.1128/CMR.00001-07. 17630334 PMC1932750

[31] Bartual SG, Seifert H, Hippler C, Luzon MA, Wisplinghoff H, Rodriguez-Valera F 2005 Development of a multilocus sequence typing scheme for characterization of clinical isolates of Acinetobacter baumannii. J Clin Microbiol 43:4382–4390. doi:10.1128/JCM.43.9.4382-4390.2005. 16145081 PMC1234098

[32] Aaron SD, Ferris W, Henry DA, Speert DP, Macdonald NE 2000 Multiple combination bactericidal antibiotic testing for patients with cystic fibrosis infected with Burkholderia cepacia. Am J Respir Crit Care Med 161:1206–1212. doi:10.1164/ajrccm.161.4.9907147. 10764313

[33] Aaron SD, Ferris W, Ramotar K, Vandemheen K, Chan F, Saginur R 2002 Single and combination antibiotic susceptibilities of planktonic, adherent, and biofilm-grown Pseudomonas aeruginosa isolates cultured from sputa of adults with cystic fibrosis. J Clin Microbiol 40:4172–4179. doi:10.1128/JCM.40.11.4172-4179.2002. 12409393 PMC139693

[34] Slinger R, Chan F, Ferris W, Yeung SW, St Denis M, Gaboury I, Aaron SD 2006 Multiple combination antibiotic susceptibility testing of nontypeable Haemophilus influenzae biofilms. Diagn Microbiol Infect Dis 56:247–253. doi:10.1016/j.diagmicrobio.2006.04.012. 16769194

[35] Saginur R, Stdenis M, Ferris W, Aaron SD, Chan F, Lee C, Ramotar K 2006 Multiple combination bactericidal testing of staphylococcal biofilms from implant-associated infections. Antimicrob Agents Chemother 50:55–61. doi:10.1128/AAC.50.1.55-61.2006. 16377667 PMC1346774

[36] Berenbaum MC 1978 A method for testing for synergy with any number of agents. J Infect Dis 137:122–130. doi:10.1093/infdis/137.2.122. 627734

[37] den Hollander JG, Mouton JW, Verbrugh HA 1998 Use of pharmacodynamic parameters to predict efficacy of combination therapy by using fractional inhibitory concentration kinetics. Antimicrob Agents Chemother 42:744–748. 9559776 10.1128/aac.42.4.744 PMC105535

[38] Bai Y, Liu B, Wang T, Cai Y, Liang B, Wang R, Liu Y, Wang J 2015 *In vitro* activities of combinations of rifampin with other antimicrobials against multidrug-resistant Acinetobacter baumannii. Antimicrob Agents Chemother 59:1466–1471. doi:10.1128/AAC.04089-14. 25534730 PMC4325818

[39] Sader HS, Huynh HK, Jones RN 2003 Contemporary *in vitro* synergy rates for aztreonam combined with newer fluoroquinolones and beta-lactams tested against gram-negative bacilli. Diagn Microbiol Infect Dis 47:547–550. doi:10.1016/S0732-8893(03)00158-5. 14596974

[40] Li J, Nation RL, Owen RJ, Wong S, Spelman D, Franklin C 2007 Antibiograms of multidrug-resistant clinical Acinetobacter baumannii: promising therapeutic options for treatment of infection with colistin-resistant strains. Clin Infect Dis 45:594–598. doi:10.1086/520658. 17682994

[41] Mendes RE, Fritsche TR, Sader HS, Jones RN 2008 Increased antimicrobial susceptibility profiles among polymyxin-resistant Acinetobacter baumannii clinical isolates. Clin Infect Dis 46:1324–1326. doi:10.1086/533476. 18444879

[42] Durante-Mangoni E, Signoriello G, Andini R, Mattei A, De Cristoforo M, Murino P, Bassetti M, Malacarne P, Petrosillo N, Galdieri N, Mocavero P, Corcione A, Viscoli C, Zarrilli R, Gallo C, Utili R 2013 Colistin and rifampicin compared with colistin alone for the treatment of serious infections due to extensively drug-resistant Acinetobacter baumannii: a multicenter, randomized clinical trial. Clin Infect Dis 57:349–358. doi:10.1093/cid/cit253. 23616495

[43] O'Hara JA, Ambe LA, Casella LG, Townsend BM, Pelletier MR, Ernst RK, Shanks RM, Doi Y 2013 Activities of vancomycin-containing regimens against colistin-resistant Acinetobacter baumannii clinical strains. Antimicrob Agents Chemother 57:2103–2108. doi:10.1128/AAC.02501-12. 23422916 PMC3632926

[44] Hornsey M, Wareham DW 2011 *In vivo* efficacy of glycopeptide-colistin combination therapies in a Galleria mellonella model of Acinetobacter baumannii infection. Antimicrob Agents Chemother 55:3534–3537. doi:10.1128/AAC.00230-11. 21502628 PMC3122470

[45] Cheng A, Chuang YC, Sun HY, Sheng WH, Yang CJ, Liao CH, Hsueh PR, Yang JL, Shen NJ, Wang JT, Hung CC, Chen YC, Chang SC 2015 Excess mortality associated with colistin-tigecycline compared with colistin-carbapenem combination therapy for extensively drug-resistant Acinetobacter baumannii bacteremia: a multicenter prospective observational study. Crit Care Med 43:1194–1204. doi:10.1097/CCM.0000000000000933. 25793437

[46] Bonapace CR, Bosso JA, Friedrich LV, White RL 2002 Comparison of methods of interpretation of checkerboard synergy testing. Diagn Microbiol Infect Dis 44:363–366. doi:10.1016/S0732-8893(02)00473-X. 12543542

