# Supplementary material for: In Vitro Synergistic Activity of Antimicrobial Agents in Combination against Clinical Isolates of Colistin-Resistant Acinetobacter baumannii
Source: Antimicrob Agents Chemother. 2016 Oct 21;60(11):6774–9. doi: 10.1128/AAC.00839-16 (PMC5075085; doi:10.1128/AAC.00839-16)
Supplement: Supplemental material [file supp_60_11_6774__index.html]

Supplemental material 

# *In Vitro* Synergistic Activity of Antimicrobial Agents in Combination against Clinical Isolates of Colistin-Resistant Acinetobacter baumannii

## Supplemental material

- Supplemental file 1 -

  Supplemental Table S1

  PDF, 26K
