# Supplementary material for: In Vitro Synergistic Activity of Antimicrobial Agents in Combination against Clinical Isolates of Colistin-Resistant Acinetobacter baumannii
Source: Antimicrob Agents Chemother. 2016 Oct 21;60(11):6774–9. doi: 10.1128/AAC.00839-16 (PMC5075085; doi:10.1128/AAC.00839-16)
Supplement: Supplemental material [file AAC.00839-16_zac011165694so1.pdf]

Table S1. Microbiological and genotypic characteristics of colistin-resistant *Acinetobacter baumannii* strains

			Type of carbapenemase			
**Strain**	**Source**	**ST**	**OXA-23**[a]	**OXA-51**	**OXA-69**	**IMP-1**
**a**	Blood	191	+	+	+	+
**b**	Blood	191	+	+	+	+
**c**	Blood	191	+	+	+	+
**d**	Blood	191	+	+	+	+
**e**	Blood	191	+	+	+	+
**f**	Blood	191	-	+	-	-
**g**	Blood	357	+	+	+	+
**h**	Blood	357	-	+	-	+
**i**	Sputum	191	+	+	+	+

[a] IS*Aba1* enhanced $\textit{bla}_{\text{OXA-23}}$-like
